# A Chemical Biology Approach to Developing STAT Inhibitors: Molecular Strategies for Accelerating Clinical Translation

**DOI:** 10.18632/oncotarget.296

**Published:** 2011-06-15

**Authors:** Erik A. Nelson, Sreenath V. Sharma, Jeffrey Settleman, David A. Frank

**Affiliations:** ^1^ Department of Medical Oncology, Dana-Farber Cancer Institute, Departments of Medicine, Harvard Medical School and Brigham and Women's Hospital, Boston, MA 02215; ^2^ Massachusetts General Hospital, Harvard Medical School; ^3^ Current address: Oncology Drug Discovery, Novartis Institutes for Biomedical Research, 250 Massachusetts Avenue, Cambridge, MA 02139

**Keywords:** STAT transcription factors, signal transduction, cancer therapy

## Abstract

STAT transcription factors transduce signals from the cell surface to the nucleus, where they regulate the expression of genes that control proliferation, survival, self-renewal, and other critical cellular functions. Under normal physiological conditions, the activation of STATs is tightly regulated. In cancer, by contrast, STAT proteins, particularly STAT3 and STAT5, become activated constitutively, thereby driving the malignant phenotype of cancer cells. Since these proteins are largely dispensable in the function of normal adult cells, STATs represent a potentially important target for cancer therapy. Although transcription factors have traditionally been viewed as suboptimal targets for pharmacological inhibition, chemical biology approaches have been particularly fruitful in identifying compounds that can modulate this pathway through a variety of mechanisms. STAT inhibitors have notable anti-cancer effects in many tumor systems, show synergy with other therapeutic modalities, and have the potential to eradicate tumor stem cells. Furthermore, STAT inhibitors identified through the screening of chemical libraries can then be employed in large scale analyses such as gene expression profiling, RNA interference screens, or large-scale tumor cell line profiling. Data derived from these studies can then provide key insights into mechanisms of STAT signal transduction, as well as inform the rational design of targeted therapeutic strategies for cancer patients.

## INTRODUCTION

The goal of research in cancer therapy is to develop treatments that specifically target the cancer cell while leaving normal cells intact. As basic scientific studies elucidate signaling pathways that are activated inappropriately in tumors and drive their pathogenesis, new therapeutic targets are emerging. One such pathway is the signal transducer activator of transcription (STAT) pathway, which allows extracellular cues to modulate gene expression [1]. Through the action of a variety of tyrosine kinases, STATs in the cytoplasm become phosphorylated on a critical tyrosine residue, thereby leading to an activating dimerization. These STAT dimers then enter the nucleus where they can modulate transcription of genes involved in key cellular processes such as survival and proliferation. Under physiological conditions, STATs are activated rapidly and transiently, reaching peak phosphorylation within minutes, and becoming dephosphorylated within one or two hours. However, in a wide range of human cancers, STATs, particularly STAT3 and STAT5, become activated constitutively, thereby driving increased expression of genes that directly lead to malignant cellular behavior [2]. Although STATs are critical for the pathogenesis of these tumors, they are largely dispensable in normal adult cells, suggesting that they would be targets with a high therapeutic index. Though transcription factors have not traditionally been thought of as druggable targets, the wide variety of cancers that depend on STATs for survival suggests that STATs may be attractive targets for cancer therapy.

## CHEMICAL BIOLOGY APPROACHES TO DEVELOPING STAT INHIBITORS

To elucidate novel pharmacological strategies to modulate STAT-dependent gene expression, we developed a non-biased screen to identify compounds that could target any part of the STAT transcriptional pathway [3]. For this chemical biology approach, we generated a series of cell lines in which a luciferase reporter gene is under the inducible control of a single transcription factor. We then used these cell lines to screen diverse chemical libraries to identify compounds that could specifically block the function of a STAT family member. One could then deconvolute the mechanism by which the identified compounds mediated their effect, and this could reveal unappreciated targets for pharmacological intervention. We then took two parallel approaches for compound screening. We interrogated large diverse libraries comprised of approximately 200,000 compounds. In addition, to accelerate the development of proof-of-concept clinical trials, we also screened libraries of drugs that were already known to be safe in humans. Using this approach, we identified nifuroxazide, which is approved in several countries for the treatment of diarrhea, as an inhibitor of STAT3. Nifuroxazide decreases STAT3 tyrosine phosphorylation, and appears to do so by inhibiting Jak family tyrosine kinases, including Jak2 and Tyk2. Reflecting the importance of this pathway in multiple myeloma (MM), nifuroxazide selectively reduces the viability of MM cells that contain constitutive STAT3 activation. Nifuroxazide has a particularly strong effect at reducing the viability of MM cells overexpressing CKS1B, which is associated with poor prognosis in MM patients and which is associated with enhanced STAT3 activation [4]. Therefore, using a non-biased approach, we have identified nifuroxazide as a STAT3 inhibitor that may be useful as a treatment for patients with MM.

**Figure 1:**
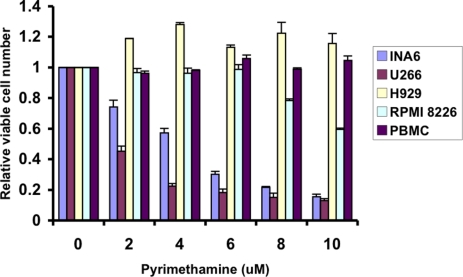
Pyrimethamine reduces the viability of multiple myeloma cell lines containing activated STAT3 Cells were incubated with the indicated concentrations of pyrimethamine for 48 hours, after which the relative number of viable cells was measured using an ATP-dependent luminescence assay. Multiple myeloma cell lines containing activated STAT3 (U266 and INA6) or lacking activated STAT3 (H929 and RPMI 8226) were tested, as were peripheral blood mononuclear cells (PBMC) harvested from healthy donors.

This screen also identified the anti-parasitic drug pyrimethamine, which is approved in the United States for the treatment of toxoplasmosis and malaria, as being an effective STAT3 inhibitor. Pyrimethamine displays significant activity in vitro against multiple myeloma cell lines characterized by activation of STAT3 (Figure 1). However, it has little effect on myeloma cell lines lacking STAT3 activation, or on peripheral blood mononuclear cells (PBMC) harvested from healthy donors, which also lack STAT3 activation. Pyrimethamine exerts at least some of its anti-microbial effects as an inhibitor of dihydrofolate reductase (DHFR). However, this is unlikely to be the sole mechanism for its effect on STAT3 signaling, as other DHFR inhibitors, such as methotrexate, did not show activity in this screen.

## STAT5 INHIBITION IN CML: PIMOZIDE

A third STAT inhibitor that we identified using this approach is the psychotropic drug pimozide, which is approved in the United States for treating Tourette’s syndrome [5]. This drug inhibits STAT5 function, and thus we performed our initial characterization in a disease in which the pathogenesis is dependent on constitutive STAT5 activation, chronic myelogenous leukemia (CML). CML is characterized by the essentially uniform finding of a chromosomal translocation between chromosomes 9 and 22 yielding a fusion kinase BCR/ABL [6, 7]. Although the introduction of kinase inhibitors such as imatinib mesylate has revolutionized the treatment of this disease, several clinical challenges persist [8]. Some patients cannot tolerate the side effects from kinase inhibitors [9], and in others mutations arise in BCR/ABL rendering it resistant to the effect of kinase inhibitors [10]. In addition, kinase inhibitors do not eradicate the leukemic stem cell, and thus patients need to take these drugs indefinitely [11]. We found that pimozide inhibits the tyrosine phosphorylation of STAT5 in CML cells, raising the possibility that it may be a BCR/ABL inhibitor. However, several lines of evidence have suggested that this is not the case. First, there is no consistent loss of BCR/ABL autophosphorylation at concentrations that lead to STAT5 inhibition. Second, though the BCR/ABL inhibitor imatinib leads to a reduction in total cellular tyrosine phosphorylation, pimozide does not affect the phosphorylation of other substrates. Third, in vitro kinase assays show no effect of pimozide on BCR/ABL activity. In addition, BCR/ABL leads to the activation of pathways other than STAT5 in CML cells. Consequently, BCR/ABL kinase inhibitors such as imatinib and nilotinib lead to a reduction in ERK MAP kinase (MAPK) phosphorylation; pimozide, by contrast, not only fails to decrease MAPK phosphorylation, but in fact leads to an increase in MAPK activation. This may be a result of a loss of expression of STAT5-dependent negative signaling regulators. Therefore, pimozide likely inhibits STAT5 phosphorylation in a BCR/ABL-independent manner. The precise mechanism by which pimozide inhibits STAT5 is unknown, but preliminary studies suggest that it may alter the activity of negative regulators of STAT5 phosphorylation.

The activation of MAPK in response to pimozide not only raises mechanistic questions, but it also provides a therapeutic opportunity. Though the inhibition of STAT5 by pimozide reduces cell survival, it is possible that the accompanying activation of MAPK by pimozide provides pro-survival signals to the cell. This raised the possibility that inhibition of kinases upstream of MAPK might be particularly beneficial when combined with pimozide. Treatment of CML cells with the MEK inhibitor UO126 reduces the phosphorylation of MAPK, and this is seen even in the presence of pimozide. The combination of pimozide and UO126 leads to an increase in cytotoxicity when compared to cells treated with either drug alone. Therefore, dissecting the effects of inhibition of signaling pathways can provide crucial insights into maximizing the therapeutic potential of this approach.

If it is true that pimozide is functioning as a STAT5 inhibitor downstream from BCR/ABL, then it would be expected that pimozide would be equally efficacious in inhibiting the viability of CML cells even in the presence of mutations in BCR/ABL that render them resistant to kinase inhibitors. Consistent with this possibility, we found that pimozide showed equal potency towards CML cells harboring wildtype BCR/ABL and BCR/ABL containing a T315I mutation which renders it resistant to all currently approved kinase inhibitors [12]. This effect may have even broader applicability in treating CML and other myeloproliferative diseases. In addition to mutations in BCR/ABL, it has been suggested that mutations in alternative pathways, such as Jak2, may result in imatinib resistance through activation of STAT5 [13-15]. A recent report suggests that the upregulation of STAT5 expression may also be part of the development of imatinib resistance [16]. All of these mechanisms require continued STAT5 function, and thus inhibitors of this protein may be able to overcome resistance in a variety of settings.

One additional effect of STAT5 inhibition emerged from these experiments. As noted, CML patients need to take kinase inhibitors indefinitely, presumably because these drugs do not eradicate the leukemic stem cell. To address this question, we examined the effect of pimozide on hematopoietic colony formation in vitro employing CD34+ cells isolated from the bone marrow of patients with CML or healthy donors. While pimozide had minimal effects on the cells from the healthy donors, it completely abrogated the ability of the cells from the CML patients to form colonies. This observation raises the possibility that pimozide or other STAT5 inhibitors, alone or perhaps in combination with a kinase inhibitor, may be able to eradicate leukemic stem cells. This is perhaps not surprising, in that some STAT target genes, such as BCL6 and KLF4, may play a critical role in maintaining pluripotency [17-19]. This is further supported by the finding that STAT3 activation may be a key event in breast cancer cells with stem cell-like properties [20].

## STAT3 AND STAT5 IN CANCER PATHOGENESIS

Although STAT3 and STAT5 have similar DNA binding sites, they have distinct biological roles. For example, in mammary epithelium, STAT5 is activated during pregnancy and lactation, while STAT3 is activated primarily during involution [21]. However, in cancer pathogenesis there is evidence that STAT3 and STAT5 have similar functions [22]. There are many genes known to be regulated by both STAT3 and STAT5, including pro-survival and proliferation genes such as Bcl-x, Mcl-1, and cyclin D1. In leukemias, STAT5 activation upregulates genes critical for tumor survival, whereas in multiple myeloma, STAT3 activates the same, or similar genes, which are critical for the pathogenesis of this tumor type. In addition, tumors that depend on the constitutive activation of one STAT may develop resistance to therapy through activation of another STAT family member. This can occur through the activation of other tyrosine kinases, through the autocrine secretion of cytokines, or through soluble or other signals derived from other cells, such as those found in the bone marrow stroma [13-15, 23]. For example, BCR/ABL activates STAT5 in the CML cell line K562, but when these cells are co-cultured with stromal cells, STAT3 is activated [24]. This activation of STAT3 is correlated with resistance to the BCR/ABL inhibitors imatinib and nilotinib. Treatment of K562 cells with imatinib leads to an inhibition of STAT5 activation and a reduction in STAT5 target gene expression. However, K562 cells co-cultured with stromal cells continue to express these STAT target genes. Furthermore, reducing STAT3 levels with siRNA reversed the imatinib resistance seen under the co-culture conditions. Therefore, it is likely that STAT3 and STAT5 can both be oncogenic by regulating the expression of critical target genes. This also suggests that STAT inhibitors may be useful as therapeutic agents in cells that contain activation of either STAT3 or STAT5, or both.

**Figure 2:**
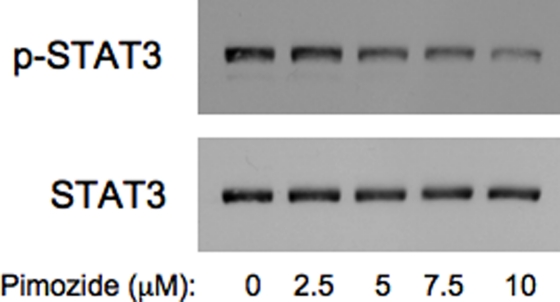
Pimozide reduces STAT3 tyrosine phosphorylation in multiple myeloma cells INA6 myeloma cells were incubated with the indicated concentrations of pimozide for three hours, after which whole cell lysates were analyzed by immunoblot for tyrosine phosphorylated STAT3 and total STAT3.

**Figure 3:**
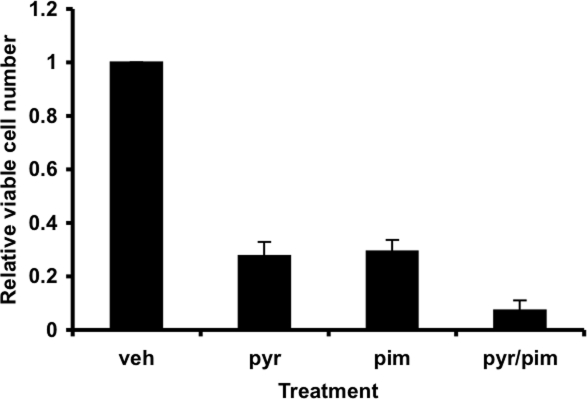
Myeloma cell viability is reduced when treated with the combination of pimozide and pyrimethamine INA6 multiple myeloma cells were treated with pyrimethamine (pyr) or pimozide (pim) or both. After 48 hours, the relative number of viable cells was measured using an ATP-dependent luminescence assay.

Since STAT3 and STAT5 share overlapping regulatory pathways, we hypothesized that pimozide might inhibit STAT3 as well as STAT5. Although there are some cell type-specific restrictions to its effects, pimozide readily inhibits STAT3 tyrosine phosphorylation in a number of systems, including the multiple myeloma cell lines INA6 (Figure 2) and U266 (data not shown). When myeloma cells are incubated with pimozide, there is a dose dependent loss of viability (data not shown). Since pimozide and pyrimethamine inhibit STAT3 through distinct mechanisms, we reasoned that they may show a beneficial effect when combined. Various dose combinations of these drugs show enhanced effects on cell viability when compared to either drug alone (Figure 3). This raises the possibility that simultaneously blocking STAT activation at two distinct steps may be a particularly effective form of therapy, and potentially less likely to allow the generation of resistance.

## LEVERAGING THE FINDINGS FROM CELL-BASED SCREENS

Having identified compounds that inhibit STATs, it then becomes possible to use these as probes to gain insight into the mechanisms of STAT signaling, identify specific targets for therapeutic intervention, and isolate even more effective drugs for therapeutic development.

### Gene expression profiling: The Connectivity Map

Although screening large numbers of compounds in a cell-based assay can rapidly identify a number of active STAT inhibitors, it can be a challenge to identify the mechanism by which these compounds exert their effect. One strategy is to identify other drugs whose mechanisms are better understood, and which exert similar actions. One approach to accomplish this is to obtain gene expression signatures from cells treated with a drug, and then to interrogate the Connectivity Map, a large database containing gene expression data derived from cells treated with over 1000 drugs [25]. This may identify other STAT inhibitors, which themselves may be useful for cancer therapy, and it may also help to clarify the mechanism of action of these drugs. It is also possible to use gene expression signatures of activated STAT3 to identify drugs that induce an inverse of this pattern as another computational strategy to identify STAT3 inhibitors.

### RNA interference screening

A second large-scale approach to understand STAT signaling more clearly and elucidate the mechanism of action of newly identified STAT inhibitors is to use siRNA-based screens in conjunction with the luciferase reporter cell lines described above. This approach can identify gene products that modulate STAT signaling, and may reveal hitherto unknown ways in which STAT signaling can be affected by other cellular pathways. RNA interference-based screens can also be combined with newly identified STAT inhibitors to identify gene products that either abrogate the effect of the drugs, or which potentiate their effect. This strategy can help identify the direct cellular targets of these compounds, which may allow more traditional drug development approaches to be applied to them. In addition, the identification of pathways that may synergize with these STAT inhibitors can immediately suggest potentially synergistic therapeutic strategies that can be developed.

### Large scale cell line-based efficacy screens

**Figure 4:**
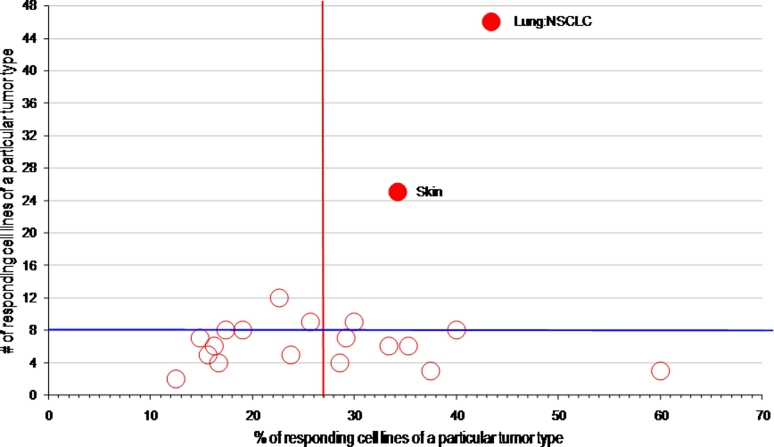
High throughput cell line profiling reveals distinct tumor type sensitivity to pyrimethamine 684 human cancer cell lines were screened for the growth inhibitory effects of pyrimethamine, tested at three different concentrations, using previously described methods [26]. The tumor type enrichment algorithm indicates cell lines derived from different tumor types that show preferential sensitivity to pyrimethamine. 27% of the cell lines tested (vertical red line) were sensitive to pyrimethamine (defined as greater than 80% killing at 10 μ M). As a threshold for activity, greater than eight distinct cell lines of a particular tumor type had to show sensitivity to pyrimethamine (horizontal blue line). Tumor types in the top right hand quadrant are significantly more sensitive to the drug and are indicated by red filled circles.

Given that STAT3 is known to be activated in a wide spectrum of human tumors, another strategy to evaluate the potential of STAT inhibitors is to determine their activity on a large panel of highly annotated cancer cell lines [26, 27]. This approach can rapidly generate information on the activity of individual compounds against cell lines derived from particular tumor types, allows for analyses based on the genotype of cell lines, and provides a comparison of the activity of distinct STAT inhibitors evaluated through such a screen. Using a high throughput system, we screened over 600 adherent cells lines for sensitivity to pyrimethamine and pimozide. Importantly, not all the cell lines were sensitive to these drugs, confirming that they are not generally cytotoxic. Pyrimethamine was particular effective against cell lines from non-small cell lung cancers and skin cancers (principally melanoma), while pimozide showed preferential effectiveness against tumors from skin, esophagus, and head and neck, among others (Figure 4 and data not shown). All of these tumors have been reported to have frequent activation of STAT3, and these findings support a common final target of these drugs [28-30]. However, the sensitivities were not superimposable, suggesting that there are drug-specific effects as well.

Since these cell lines have been genetically characterized, they can provide other clues regarding the effects of these drugs. For example, activating mutations in BRAF have been found to occur commonly in melanoma [31], and pimozide is more effective in tumors containing this mutation. Just as pimozide shows enhanced efficacy in CML when combined with BCR/ABL inhibitors, pimozide may be particularly useful in melanoma when combined with BRAF inhibitors [32, 33]. In addition, pimozide appears to be more effective in tumors that contain wildtype p53, which raises both mechanistic questions regarding STAT3 inhibitors in general and these drugs in particular. These findings also suggest potential therapeutic strategies that could be employed to optimize the effects of these agents clinically. Thus, the identification of targeted STAT inhibitors through chemical biology processes can be leveraged by these additional large-scale analyses.

## CONCLUSION

Since the identification of the STAT transcription factor family in the early 1990s, abundant evidence has linked the inappropriate activation of these proteins with cancer pathogenesis. Given that these proteins may be relatively dispensable in mature cells, they represent targets with the potential of having a high therapeutic index. Although transcription factors have traditionally been viewed as difficult targets for pharmacological inhibition, chemical biology approaches have been useful in identifying compounds that can specifically inhibit these proteins through a variety of mechanisms. Furthermore, large scale strategies based on gene expression analysis, RNA interference, and cytotoxicity assays can then provide additional information related to both basic questions in STAT signal transduction, as well as the development of rational therapeutic strategies for cancer patients.
